# Toward a Translational Model of Sex‐Associated Pavlovian Phenotypes

**DOI:** 10.1111/adb.70054

**Published:** 2025-06-02

**Authors:** Luigi A. E. Degni, Sara Garofalo

**Affiliations:** ^1^ Department of Psychology University of Bologna Cesena Italy; ^2^ International School of Advanced Studies University of Camerino Camerino Italy

## Abstract

A recent study by Hakus et al. (2025) demonstrated sex‐associated differences in Pavlovian phenotypes in rodents, with females more likely to exhibit sign‐tracking behaviour and males more likely to exhibit goal‐tracking behaviour. In the present work, we provide evidence that similar patterns emerge in humans. Using a validated eye‐tracking procedure in a Pavlovian learning paradigm, we show that women are more frequently classified as sign‐trackers and quantitatively show greater sign‐tracking behaviour than men in a large human sample. These results support the translational value of preclinical findings and highlight the importance of considering sex differences in incentive salience attribution. Given the established link between sign‐trackers and addiction vulnerability, our findings may help refine our understanding of individual risk factors in the development of such disorders.

## Graphical abstract

Using a validated eye‐tracking procedure within a Pavlovian conditioning paradigm in a large human sample, we replicate rodent findings showing that females are more likely to exhibit sign‐tracking behaviour, whereas males more often display goal‐tracking behaviour. These findings highlight the translational value of animal models and suggest that sex‐related differences in incentive salience attribution may play a key role in addiction vulnerability.

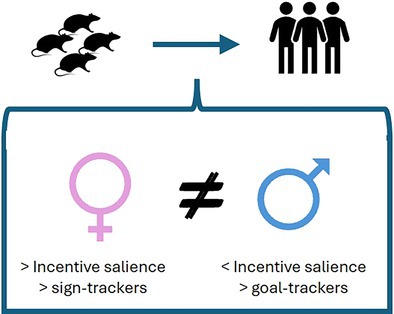

A recent study published by Hakus et al. [1] in Addiction Biology investigated sex‐associated differences in incentive salience and alcohol consumption in rodents. In their study, incentive salience attribution was measured through a Pavlovian conditioned approach (PCA) paradigm, which provides a framework for distinguishing between two behavioural phenotypes related to the motivational value assigned to reward‐predictive cues (i.e., conditioned stimuli [CSs]). Specifically, when CS and reward are presented in different locations, at the CS presentation, some animals—known as goal‐trackers—primarily interact with the reward location, while others—known as sign‐trackers—approach the CS. Notably, while both groups recognize the predictive value of reward‐predictive cues, sign‐trackers assign greater incentive salience to them, a characteristic that has been widely linked to increased vulnerability to addiction. Their findings demonstrate that female rats are more likely to exhibit sign‐tracking behaviour compared to males, and that this phenotype correlates with higher alcohol intake. The reported results align with the observations made by Hughson et al. [2], who found similar sex differences in sign‐tracking behaviour using a PCA paradigm on a large rodent sample. Furthermore, they confirm previous research indicating that the reinforcing effects of alcohol are more pronounced in female rats than in males [3]. In summary, the study performed by Hakus et al. confirms once again the importance of preclinical research for unravelling the complex relationship between motivation, clinical disorders and biological sex.

However, an important open question remains, do similar sex‐based patterns in behavioural phenotypes emerge in humans? The translational potential of these findings would be crucial for understanding addiction vulnerability and possibly preventing the development, maintenance and relapse of this and other reward‐ and impulsivity‐related disorders in humans. Yet this raises the issue of whether humans might be categorized as sign‐ or goal‐trackers using analogous criteria. The challenge with human experimental settings is that direct measures of behavioural approach (as done in the PCA paradigm) are difficult to implement [4]. Nevertheless, Garofalo and di Pellegrino [5] first demonstrated that sign‐tracking and goal‐tracking behaviours can be determined in humans by measuring oculomotor responses. In their experiment, the authors developed a Pavlovian conditioning procedure in which CSs (i.e., ‘signs’) were presented one at a time on a computer screen, and followed by rewards (i.e., ‘goals’) appearing in different locations. Eye gaze was measured through eye‐tracking to mimic the approach behaviour observed in PCA. The classification occurred when only the CS was presented on the screen: participants who spent more time looking at the CS were defined as sign‐trackers, while participants who spent more time looking at the (still empty) location where subsequently the reward would appear were defined as goal‐trackers.

This paradigm (or slightly modified versions) has been used over the past few years to test several aspects of sign‐tracking and goal‐tracking behaviours and to confirm the putative association between sign‐trackers and maladaptive behaviours in humans (see [6] for a review). These experiments further validated the paradigm by demonstrating its ability to effectively distinguish between sign‐trackers and goal‐trackers while confirming most of the behavioural and neural characteristics associated with these two phenotypes in animal models.

Here, we merged data from multiple, although yet unpublished, recent studies from our lab to test whether Hakus and colleagues' predictions for rodent models hold in a large human sample (*N* = 232; 119 females). In these experiments, the eye gaze index during a Pavlovian conditioning procedure as described above, was calculated based on the dwell time spent on the CS location minus the dwell time spent on the reward location, divided by the sum of the dwell time spent on the CS location, the reward location and the background (see Figure 1a; for the complete task specifications, refer to [7]). Therefore, a gaze index of 1 indicated that the entire time was spent looking at the CS, while −1 indicated that the entire time was spent looking at the reward. In other words, the closer the gaze index to 1, the stronger the sign‐tracking behaviour. Based on this index, participants were divided into three groups: the top third were classified as sign‐trackers, the middle third as intermediates and the bottom third as goal‐trackers. Despite our procedure included both CS+ and CS− trials, only CS+ trials (i.e., trials in which a stimulus predictive of a reward was presented) were considered for the classification in sign‐ and goal‐trackers. Eye‐gaze index during CS+ and CS− trials were then compared to ensure the absence of a general attentional bias. The results revealed a striking similarity to those reported in rodents: a greater proportion of women were classified as sign‐trackers, while men were more frequently classified as goal‐trackers (χ^2^_(2)_ = 8.62, *p* = 0.013; see Figure 1b). Moreover, we conducted an independent sample *t*‐test using eye gaze index as dependent variable, and sex (2 levels: male, female) as independent variable, which confirmed that women exhibited more sign‐tracking behaviour compared to men (t_(230)_ = 2.2, *p* = 0.029, d_cohen_ = 0.29; see Figure 1c). Importantly, to confirm that our effect did not reflect a general (or sex specific) attentional bias toward the upper or the lower side, we performed a mixed‐effect 2 × 2 ANOVA using the eye gaze index as dependent variable, the type of CS (2 levels: CS+, CS−) as within‐subjects independent variable, and sex (2 levels: male, female) as between‐subjects independent variable. Results showed a statistically significant main effect of the CS (F_1,230_ = 8.6; *p* = 0.004; η_p_^2^ = 0.4), which reflected an increased eye gaze index to CS+ (M = 0.6, SD = 0.36) than to CS− (M = 0.55, SD = 0.33). In contrast, the main effect of sex (F_1,230_ = 2.36; *p* = 0.13; η_p_^2^ = 0.01) and the sex by CS interaction (F_1,230_ = 0.13; *p* = 0.72; η_p_^2^ = 0.01) were not statistically significant. Overall, these results confirmed increased eye gaze index to salient motivational stimuli regardless of sex, thus excluding the possibility of a general attentional bias at the basis of the observed effect.

**FIGURE 1:**
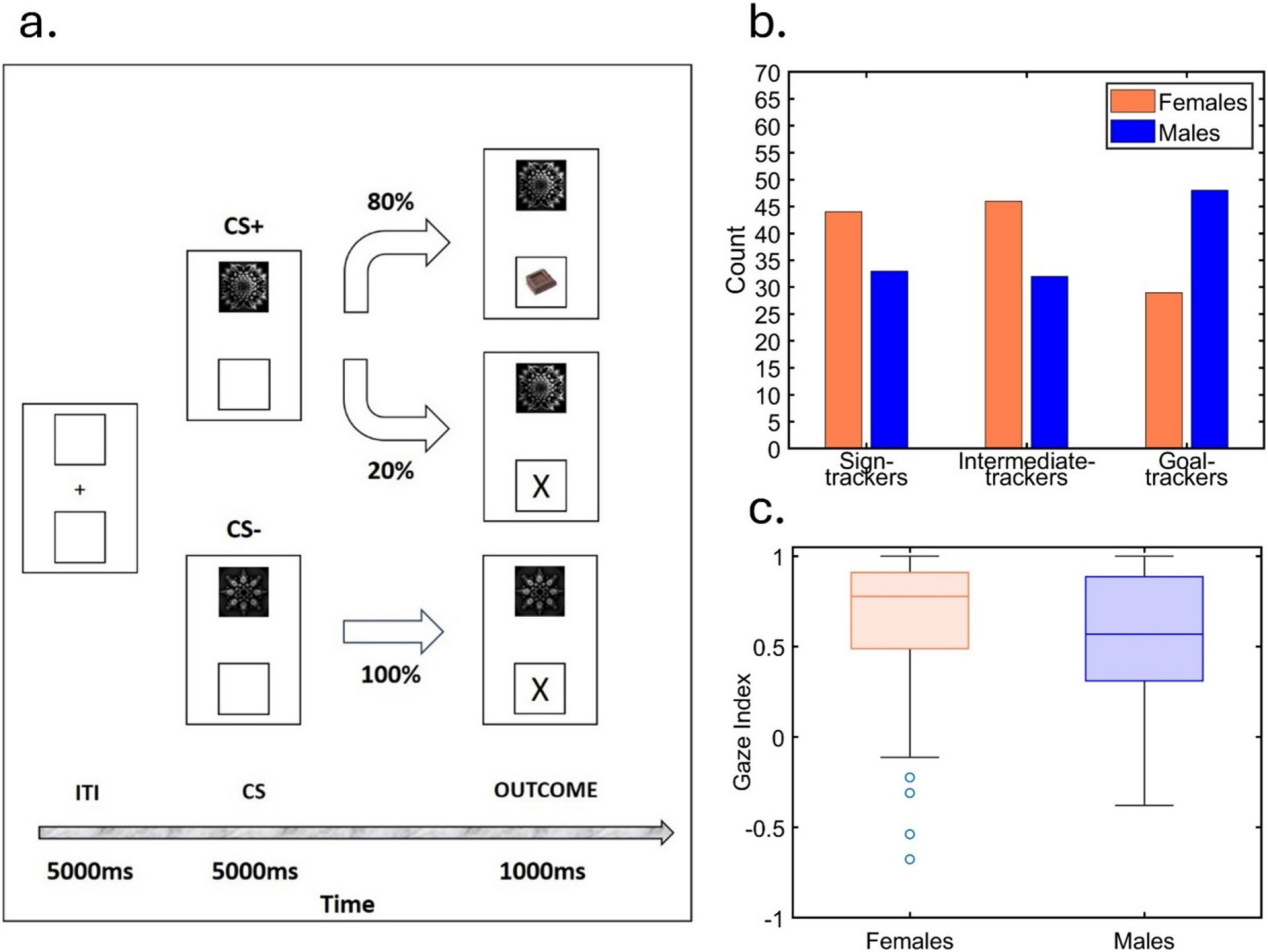
(a) Schematic outline of the Pavlovian conditioning paradigm. After an intertrial interval (ITI) of 5 s, a conditioned stimulus (CS) appeared in the upper display for 5 s. Participants were categorized as sign‐trackers, intermediate or goal‐trackers during these 5 s. CS+ was followed for 1 s by a reward (80% of trials) or a no‐reward (‘x’ in 20% of trials). CS− was always followed by no‐reward (‘x’ in 100% of trials). The classification occurred only in the last 4 s (the first second was excluded to eliminate the orienting response) of the CS+ trials in the second half of the task, i.e., when contingencies were more learned. Full methods can be found in [7]. (b) Histogram of the number of sign‐trackers, intermediate and goal‐trackers in males (blue) and females (orange). The plot shows a higher proportion of females among sign‐trackers and intermediate‐trackers, whereas males are more prevalent among goal‐trackers. (c) Boxplots showing eye gaze index in females (orange) and males (blue). A statistically significant higher gaze index was found in females than in males. Analyses performed excluding the four female outliers gave the same results (t_(226)_ = 3.24, *p* = 0.001, d_cohen_ = 0.43).

Overall, we demonstrated the translational value of Hakus and colleagues' results in humans concerning sex prevalence in incentive salience attribution. Unfortunately, our experiments did not assess alcohol‐drinking or similar behaviours, so we cannot comment on potential sex‐related differences in addiction‐related processes. Previous findings recommend caution in making overstated claims about the link between sex, sign‐trackers, and addiction in humans. Indeed, although some preliminary evidence suggests a stronger sign‐tracking behaviour in impulsivity and addictive disorders [5, 8], opposite patterns have also been reported in recent years [9]. Moreover, Hakus and colleagues themselves reported conflicting findings regarding the epidemiological prevalence of addiction in males and females, and our previous research also indicated higher addiction‐related behaviours in males [10]. A possible explanation is that addiction vulnerability is affected by the interaction between sex and behavioural phenotypes in Pavlovian conditioning, and future studies should consider separately males and females sign‐trackers. In conclusion, despite plenty of open questions remain, our findings can be a first step toward a unitary and translational model for sign‐ and goal‐trackers in addiction vulnerability, which should consider sex as a crucial variable to predict addiction trajectories over time.

## Conflicts of Interest

The authors declare no conflicts of interest.

## Data Availability

The data that support the findings of this study are available from the corresponding author upon reasonable request.
